# Analysis of induced electrical currents from magnetic field coupling inside implantable neurostimulator leads

**DOI:** 10.1186/1475-925X-10-94

**Published:** 2011-10-21

**Authors:** Oxana S Pantchenko, Seth J Seidman, Joshua W Guag

**Affiliations:** 1 U.S. Food and Drug Administration, 10903 New Hampshire Avenue, Silver Spring, MD 20993, USA; 2 University of California at Santa Cruz, Baskin School of Engineering, 1156 High Street, Santa Cruz, CA 95064, USA

## Abstract

**Background:**

Over the last decade, the number of neurostimulator systems implanted in patients has been rapidly growing. Nearly 50, 000 neurostimulators are implanted worldwide annually. The most common type of implantable neurostimulators is indicated for pain relief. At the same time, commercial use of other electromagnetic technologies is expanding, making electromagnetic interference (EMI) of neurostimulator function an issue of concern. Typically reported sources of neurostimulator EMI include security systems, metal detectors and wireless equipment. When near such sources, patients with implanted neurostimulators have reported adverse events such as shock, pain, and increased stimulation. In recent *in vitro *studies, radio frequency identification (RFID) technology has been shown to inhibit the stimulation pulse of an implantable neurostimulator system during low frequency exposure at close distances. This could potentially be due to induced electrical currents inside the implantable neurostimulator leads that are caused by magnetic field coupling from the low frequency identification system.

**Methods:**

To systematically address the concerns posed by EMI, we developed a test platform to assess the interference from coupled magnetic fields on implantable neurostimulator systems. To measure interference, we recorded the output of one implantable neurostimulator, programmed for best therapy threshold settings, when in close proximity to an operating low frequency RFID emitter. The output contained electrical potentials from the neurostimulator system and those induced by EMI from the RFID emitter. We also recorded the output of the same neurostimulator system programmed for best therapy threshold settings without RFID interference. Using the Spatially Extended Nonlinear Node (SENN) model, we compared threshold factors of spinal cord fiber excitation for both recorded outputs.

**Results:**

The electric current induced by low frequency RFID emitter was not significant to have a noticeable effect on electrical stimulation.

**Conclusions:**

We demonstrated a method for analyzing effects of coupled magnetic field interference on implantable neurostimulator system and its electrodes which could be used by device manufacturers during the design and testing phases of the development process.

## Background

Radio frequency identification (RFID) readers are being used for tracking people, animals, products and goods. Some of the advantages of this technology include proximity of identification and robust ability to store information. RFID readers can identify tags within meters away. This technology works by emitting and receiving radio frequency electromagnetic energy. Since RFID technology has gained popularity in many industries, an average person could get exposed to the emitted fields from RFID readers when using public transportation, shopping at a grocery store, picking up a package at a postal service and driving through a toll booth [1,2].

The United States Food and Drug Administration (FDA) is encouraging use of a state-of-the-art technology, such as RFID, to allow manufacturers and distributors to precisely track drug products through the supply chain [3]. Overall, RFID systems offer a variety of benefits, including fast transactions, real time tracking, contactless data transfer, large storage capacity and continuous temperature monitoring. Some claim that RFID technology can change the delivery of patient care [4].

In recent years, the FDA has received a series of adverse event reports suggesting electromagnetic interference (EMI) with deep brain and spinal cord stimulators from various electromagnetic sources [5]. Kainz *et. al*. reported various sources of EMI, which included a report from a patient with an implantable spinal cord stimulator who received an electric shock while walking near an article surveillance device [6]. Several incident reports and published literature culminated in the 1998 FDA advisory to cardiologists, cardiovascular surgeons, emergency physicians, neurologists and neurosurgeons warning that the operation of certain medical devices, including spinal cord stimulators, may be affected by the electromagnetic fields produced by anti-theft systems and metal detectors [7].

In our recent study, we investigated the EMC of six implantable neurostimulators and 22 RFID emitters. We found effects of inhibition of one implantable neurostimulator system indicated for incontinence when close to two low frequency RFID emitters. The effects were determined to be clinically significant only if they occurred for extended periods of time. There were no observed effects on the other 5 implantable neurostimulators or during exposures from other RFID emitters [8]. Another publication reported that magnetic fields can turn the stimulation on or off and varying magnetic fields can momentarily inhibit telemetry [9].

A previously published study has shown that magnetic resonance systems are capable of generating strong magnetic fields that could potentially cause nerve stimulation. The induced fields inside the patients with implantable leads are much larger than induced fields inside patients with no implantable systems [10]. To address the concerns posed by coupling of electromagnetic fields from RFID emitters, we developed a test protocol to assess the interference from coupled magnetic fields on implantable neurostimulator systems.

### Background Theory

In the 1830s, Michael Faraday proposed a hypothesis that a magnetic field could produce an electric current in a wire. After 10 years of experiments, Michael Faraday and Joseph Henry independently discovered that magnetic fields can produce an electric current in a closed loop, but only if magnetic flux linking the surface area of the loop changes with time. This type of process was named electromagnetic induction. The electric current can be generated under any one of the three conditions:

1. A stationary loop in a time varying magnetic field,

2. A moving loop with a time varying area in a static magnetic field, or

3. A moving loop in a time varying magnetic field [11].

One hundred and eighty years later, the same theory still holds. This relation is now known as Faraday's law of induction.

In our work, we chose to focus on the first example of a stationary loop in a time varying magnetic field since it represents the worst case scenario of magnetic fields coupling at the minimum separation distance. In our study, the time varying magnetic field is generated by the RFID emitter and a stationary loop is formed by the implantable neurostimulator leads.

In the case of a single turn, conducting circular loop with surface area A in a time varying magnetic field B(t), the electrical potential difference between the ends of leads is defined as,

V = ∫ ∂∕∂tB⋅dA

where,

V: electrical potential difference (V)

∂/∂t **B**: rate of change of the magnetic field (T/s)

A: surface area of the enclosed loop (m^2^) [11]

## Methods

### Probe for measuring electrical potential difference

**Figure 1:**
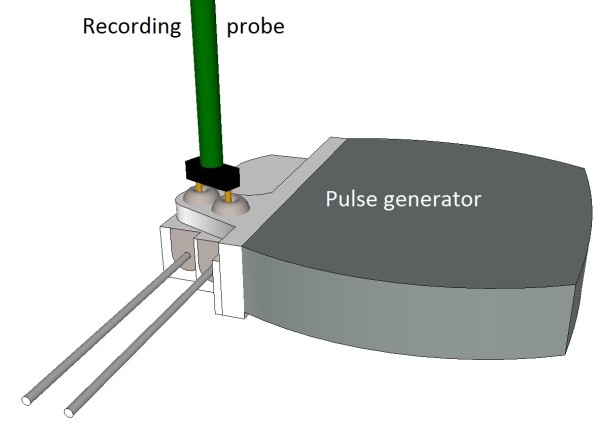
**Recording probe and pulse generator with leads attached**.

We designed a probe for measuring the electrical potential difference between two contacts of the pulse generator. The probe was made of solid conductor copper wires of American Wire Gauge 26 and a "U" shaped custom modified connector. Two pieces of copper wire were twisted and soldered to the "U" shaped custom modified connector. The connector's bare tips were separated by 5 mm and were approximately 5 mm long. The dimensions of this probe were defined by the design of the implantable neurostimulator system. The probe was designed to make contact with a set of screws that secure leads inside the pulse generator can. Figure 1 demonstrates such set up. The proximal end of the probe was connected to 1 Mega Ohm input impedance oscilloscope. The probe was oriented in a straight line parallel to the **B **field generated by the RFID antenna and perpendicular to the neurostimulator leads.

### RFID Emitter

A commercially available RFID system was used for exposing the implantable neurostimulator system to magnetic fields. The system operated using International Organization for Standardization 11785 Standard. The carrier frequency was 134.2 kHz, which is considered low frequency, or LF RFID. Typically, systems with low carrier frequency are used in access control of animals and people [12]. The maximum magnetic field strength was measured to be 269 A/m at 2.5 cm away from the RFID antenna. The pulse repetition rate of this system is 10.7 Hz. The RFID reader antenna dimensions are 20 cm × 20 cm × 2.5 cm. The highest magnetic field measured at close distances was near the corners.

### Implantable Neurostimulator System

A commercially available implantable neurostimulator system was analyzed for coupling of electromagnetic fields. This system was previously approved by the FDA for intended use of pain relief. The system consisted of an implantable pulse generator and two implantable leads with platinum/iridium electrodes. Each lead was measured to be 75 cm long with 8 electrode contacts. The system operated as an open loop system and did not require a physiological signal for activation. The unique design of the pulse generator, that enabled us to record from the electrodes, allowed us to perform the tests without modifying the pulse generator or leads.

### Patient Simulating Tank

We used a commercially available phantom ELI4, SPEAG. The phantom was made from vinylester, glass fiber reinforced material which is capable of holding up to 30 liters of fluid. The inside of the phantom has oval shape of 40 cm × 60 cm with the 2 mm bottom plate. The phantom was filled with saline solution of electrical conductivity that represents electrical properties of the body [13].

### SENN Model

The Spatially Extended Nonlinear Node (SENN) model was developed by J. Patrick Reilly and Alan M. Diamant and recently published in Electrostimulation; Theory, Applications, and Computational Model for modeling various nerve and muscle fibers [14]. The SENN model is based on a program developed by Donald McNeal in 1976. Reilly and Diamant have successfully employed this model in studying excitable tissue reactions to applied electrical forces. The SENN model accounts for temporal and spatial variations of electrical current in spinal cord fiber which includes variations in stimulus wave shape, duration, repetition pattern, magnitude, means of delivery at location of electrodes on the body, electrode size and biological and physiological factors. This model was previously used in analyzing medical applications of electro-stimulation, electric shock, exposure limits to patients in medical applications, electromagnetic safety standards and human reactions to electric weapons [14]. We implemented this model in analyzing coupling of electromagnetic fields when in close proximity to RFID emitters. The SENN model source code, supporting files, sample executable for PC and Mac platforms and a startup guide are available as free downloads at http://www.artechhouse.com/static/reslib/reilly/reilly.html.

### Procedure

**Figure 2:**
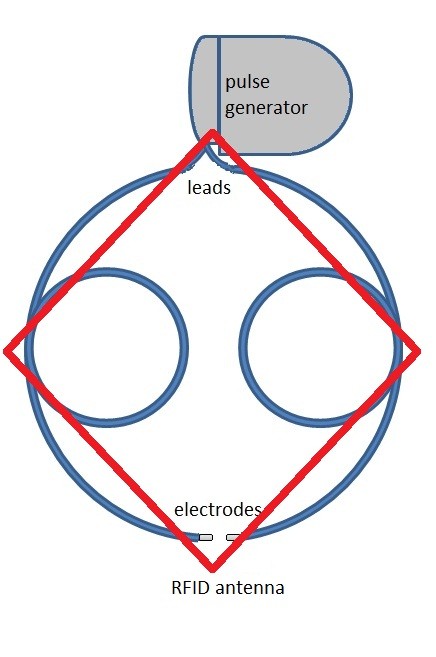
**Implantable Neurostimulator System configuration**. Top View. Each neurostimulator lead is 75 cm long, forming circular loops along RFID antenna. Assume the neuron submerged and is oriented perpendicular to the electrodes.

**Figure 3:**
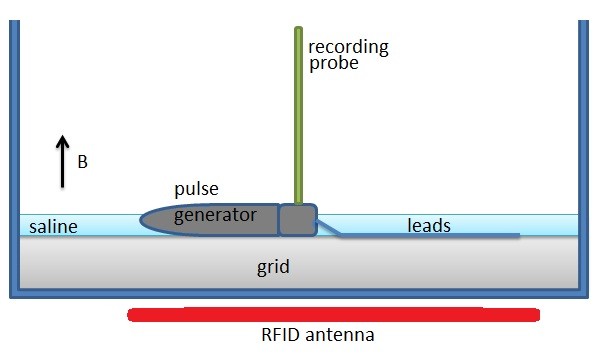
**Implantable neurostimulator system configuration inside patient simulation tank with RFID antenna shown underneath the tank**. Side View. The neuron is submerged 1.5 mm and is oriented perpendicular to the electrodes.

A non-conductive, non-metallic plastic grid was used as a support grid for the neurostimulator device and the lead system. The height of the grid was 7.7 mm. The plastic grid with the neurostimulator system attached was placed inside the patient simulation tank at the bottom. The tank was filled with 5280 μS/cm (0.528 S/m) conductivity saline to the top surface of the neurostimulator pulse generator. The conductivity value was selected to match reported 561 Ohm resistance [15] between electrodes which were 6 mm apart. The holes in the grid filled with the surrounding saline. Figures 2 and 3 demonstrate top and side views of the experimental set up.

The neurostimulator system was configured so that the leads outline the RFID antenna and the extra length was looped around the outline of the corners of the antenna (Figure 2). The magnetic field around the corners of the antenna was previously recorded to generate the highest magnetic field strengths. This layout represents the worst case scenario for coupling electromagnetic fields into implantable leads. The worst case scenario layout was also verified experimentally.

The neurostimulator system was programmed to measure the output impedance between most distal electrodes of each lead. At the separation distance between edges of electrodes of 6 mm and saline conductivity of 0.5280 S/m, the measured impedance was 561 Ohms. This impedance value was within reported values for spinal cord stimulators with dual lead configuration of 562 ± 389 Ohms [15].

Neurostimulator parameters were chosen from a recent study that reported electric parameters optimized for spinal cord stimulation with conventional non-rechargeable neurostimulator systems, with a sample size of 73 systems. The average reported parameters for best therapy were; amplitude of 5.3 mA, pulse width of 300 μsec and pulse repetition of 100 Hz (10 msec) [15]. Since our implantable neurostimulator system was a voltage controlled system, we calculated 5.3 mA (through 561 Ohms impedance) to be 3 V. We programmed the neurostimulator system to output a waveform of 3 V amplitude, 300 μsec pulse width and 100 Hz repetition rate.

Next, we connected our recording probe to the pulse generator. Using the oscilloscope, we verified programmed parameters and recorded the waveform. The same procedure was then performed three times to show repeatability.

We placed the RFID antenna underneath the saline-filled neurostimulator tank. The antenna was oriented parallel to the loop formed by the neurostimulator system's leads and perpendicular to the recording probe. Figure 3 demonstrates the side view of the layout. While the neurostimulator system was on, we turned on the RFID emitter. On the oscilloscope, we measured and recorded the output waveform which contained the waveform generated by the neurostimulator system and also the induced voltage coupled into the implantable neurostimulator leads. The same procedure was then performed three times to show repeatability.

The recorded data was then corrected for direct current (DC) coupling, possibly causing an offset that was not balanced out. This particular type of offset is considered to be caused by the recording set of instruments. The offset was determined by averaging a non-signal portion of the data and the bias was subtracted from all sample values [14].

The SENN model was then used to analyze corrected data to compile threshold factors for excitation of spinal cord fiber. The SENN model required a set of temporal and spatial parameters. We obtained spinal cord fiber standard parameters for recruitment of dorsal column fibers in spinal cord stimulation from previously published work by Struijk *et. al*. [16]. Table 1 demonstrates the parameters in further detail. For spatial parameters, we considered the worst case scenario (the shortest distance between electrode and the nerve fiber) along with optimum electrode geometry published by Holsheimer and Wesselink [17]. Table 2 defines spatial parameters in further details.

**Table 1:** Spinal Cord Fiber Standard Parameters for Recruitment of Dorsal Column Fibers in Spinal Cord Stimulation [16]

Parameters	Values
diameter of main fiber	6 μm
nodal length	1.5 μm
intra axonal resistivity	0.7 Ohm m
medium resistivity	0.58 Ohm m
membrane conductivity	1280 Ohm^-1 ^m^-2^
membrane capacitance per unit area	0.02 F m^-2^

**Table 2:** Spatial Parameters in Spinal Cord Stimulation

Symbol	Parameters	Values (cm)
Xc	x-coordinate of cathodic electrode	0.0
Yc	y-coordinate of cathodic electrode	0.15^17^
Xa	x-coordinate of anodic electrode	0.6
Ya	y-coordinate of anodic electrode	0.15^17^
Wire_L_	wire electrode length	0.85
FS	spatial field source	wire electrodes
S	electrode environment	electrodes on surface

## Results

**Figure 4:**
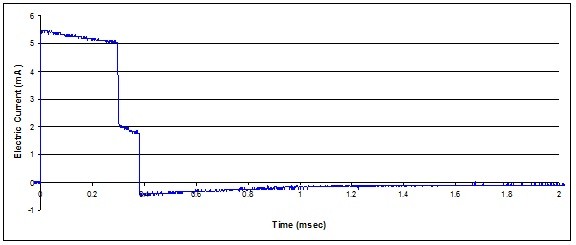
**Recorded implantable neurostimulator output waveform**.

**Figure 5:**
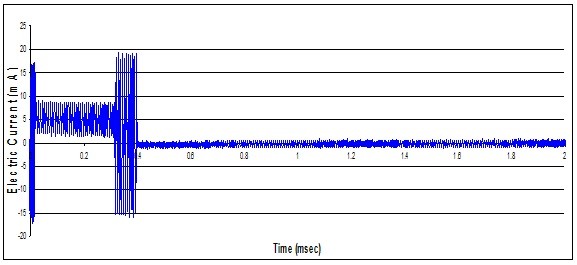
**Recorded implantable neurostimulator output waveform when in close proximity to RFID emitter**.

We recorded two types of output waveforms from the implantable neurostimulator system. The first type of waveform contained only voltage signals from the implantable neurostimulator system. The second type of waveform contained voltage signals generated by the implantable neurostimulator and induced voltage caused by EMI from the RFID emitter. Figures 4 and 5 show the recorded waveforms respectively.

Next, we generated a time-ordered digital array of (x, y) pairs for each recorded output waveform. In the SENN model, we used the sampled temporal waveform option, IWAVE 13, which is commonly used to compute thresholds of temporal waveforms recorded from electrical devices. In our case, we used this model to compute threshold factors for excitation of spinal cord fiber. In order to process an array of sampled date, the SENN model traces the waveform by indexing through the array and reading (x, y) pairs [14].

We recorded the output waveform from implantable neurostimulator three times; the SENN model generated three threshold multipliers of identical values, which were 0.22198. The reciprocal of 0.22198 is 4.5; this means that the waveform is 4.5 times larger in amplitude than needed to generate an action potential in spinal cord fiber. The reciprocal of a SENN threshold multiplier is also knows as a "threshold factor" or the ratio of applied stimulus to stimulus at threshold of excitation.

Additionally, we recorded three output waveforms from implantable neurostimulator with induced voltage caused by the RFID emitter. The SENN model generated three threshold multipliers, 0.2304, 0.2304 and 0.2264. The corresponding threshold factors are 4.34, 4.34 and 4.42 respectively. This means that the recorded waveform is 4.34 and 4.42 times larger in amplitude than needed to generate an action potential. We expected all reciprocals of threshold multipliers to be greater than unity in amplitude because the recorded waveforms were of parameters to stimulate a number of nerve fibers at a time.

Furthermore, one of the objectives of this study was to determine the severity of EMI caused by an RFID emitter. From the obtained multiplier thresholds, the results show that a stimulation pulse with EMI from an RFID emitter has an effect that is equivalent to a smaller in amplitude stimulating waveform. We did further investigation and determined that the threshold factor of 4.34 in SENN model is equivalent to 5.1 mA and 300 μsec output waveform from a spinal cord neurostimulator. Referring back to the study where we obtained best therapy parameters for stimulation [15], the study additionally lists a perception threshold of 4.6 mA in amplitude and 300 μsec in pulse width and a discomfort threshold of 6.0 mA in amplitude and 300 μsec in pulse width. Overall, the output waveform with amplitude of 5.1 mA is higher than the perception threshold and lower than discomfort threshold.

## Discussion

Overall, we recorded waveforms with and without induced voltage from RFID emitter. The waveforms with induced voltage from RFID emitter received smaller threshold factors than recorded waveforms without the induced voltage. This could be due to the dominant set of waveforms generated by the neurostimulator system itself. We referred to published in literature on strength-duration relationships derived from the myelinated nerve models. The electric current thresholds for single-cycle biphasic stimuli with an initial cathodic phase and a point electrode that is 2 mm distant from 20 μm fiber are as follows. The current threshold for 300 μsec pulse width biphasic pulse is approximately 0.6 mA, while the current threshold of 7.46 μsec (low frequency RFID pulse of 134 kHz) sine-wave pulse is approximately 40 mA [[18], Figure 4.16]. The RFID pulse of 134 kHz in frequency is signification higher in frequency than pulses generated by spinal cord neurostimulator system and therefore must be much higher in amplitude in order to generate an action potential.

In our case, the signal generated by the neurostimulator was set at 5.3 mA peak, which is larger than the reported 0.6 mA peak. The RFID interference was recorded at approximately 18 mA, which is below the needed amplitude of 40 mA to generate an action potential. When combined, in this case, the RFID interference caused a subtractive effect; the dominant waveform is minimized and therefore causes a slight reduction in threshold factors. This could be due to the way the neurostimulator system is designed to generate waveforms. In Figures 5, the period between 400 μsec and 2 msec, the interference recorded is at the minimum. This could indicate that the electrodes are connected to a high resistance inside the pulse generator. During switching times, between 0-20 μsec and 300-400 μsec of Figure 5, the interference is recorded at its maximum; this indicates that the electrodes are connected to a low impedance load inside the pulse generator. Between the times of 20 μsec and 300 μsec or when the pulse generator is outputting the monophasic pulse, it is not very clear how electrodes are connected internally without knowing the circuitry used for generating waveforms.

If the neurostimulator system was designed differently, for example having a low impedance connection between electrodes during the pulse output off period, this would cause a significantly greater voltage across electrodes in saline, therefore causing a significant addition to the output waveform that could potentially have an additive effect on the stimulation threshold factor, possibly bringing stimulation parameters to discomfort level.

## Limitations

The study was conducted on a phantom filled with saline solution representing the average conductivity of a spinal cord. The spinal cord is a complex structure consisting of various layers of nerve fibers, gray matter, white matter, cerebrospinal fluid and epidural fat; the electrical conductivities of which could vary in three orders of magnitude. Changes in electrical conductivities could results in changes of electrical current supplied by the neurostimulator system. The neurostimulator lead was configured for the worst case scenario for the specific RFID antenna used in this study. The worst case scenario represents the maximum coupling of electromagnetic fields into the neurostimulator system leads and therefore the largest induced voltages. Such a configuration does not represent the way such device would most likely be implanted. In addition, the SENN model was used to analyze output waveforms of a single spinal cord fiber. We applied this model to the best therapy stimulation parameters for one specific nerve fiber and not to all fibers in the spinal cord.

## Conclusion

From the results of our analysis, we concluded that electric currents induced by coupling of magnetic fields from a LF RFID emitter to the leads of a neurostimulator do not bring the best therapy stimulation parameters below perception threshold stimulation parameters or above discomfort threshold stimulation parameters in one implantable neurostimulator system indicated for pain relief. Overall, we demonstrated a method for analyzing effects of coupled magnetic field interference on implantable neurostimulator systems and its electrodes which could be used by device manufacturers during the design and testing phases of the development process. This method could also be applied to other implantable devices such as pacemakers, implantable cardioverter defibrillators and other types of active implantable devices. Manufacturers of active implantable devices need to be aware of the potential risk of EMI from RFID emitters and design their medical devices appropriately. Additionally, RFID industry should take into account the potential effects on active implantable medical devices when designing, configuring and installing their systems. Moreover, patients and physicians should all be aware of the possibility of adverse effects of implantable neurostimulators from RFID emitters. In the future, our goal is to increase the number and variety of tested implantable neurostimulators.

## Competing interests

The authors declare that they have no competing interests.

## Authors' contributions

OSP, SJS and JWG have made substantial contributions to experiment conception, design, acquisition, analysis and interpretation of data. All authors read and approved the final manuscript.
